# Professional Decision-Making in Research (PDR): The Validity of a New Measure

**DOI:** 10.1007/s11948-015-9667-8

**Published:** 2015-06-14

**Authors:** James M. DuBois, John T. Chibnall, Raymond C. Tait, Jillon S. Vander Wal, Kari A. Baldwin, Alison L. Antes, Michael D. Mumford

**Affiliations:** Division of General Medical Sciences, Washington University School of Medicine, 4523 Clayton Avenue, Campus Box 8005, St. Louis, MO 63110 USA; Department of Neurology and Psychiatry, Saint Louis University School of Medicine, Monteleone Hall, 1438 S. Grand Boulevard, St. Louis, MO 63104 USA; Department of Psychology, Saint Louis University, 3700 Lindell Boulevard, Morrissey Hall, St. Louis, MO 63108 USA; Department of Psychology, University of Oklahoma, 455 W. Lindsey Street, Dale Hall Tower, Norman, OK 73019 USA

**Keywords:** Research ethics, Responsible conduct of research, Research integrity, Educational, Assessment, Measurement, Professionalism

## Abstract

In this paper, we report on the development and validity of the Professional Decision-Making in Research (PDR) measure, a vignette-based test that examines decision-making strategies used by investigators when confronted with challenging situations in the context of empirical research. The PDR was administered online with a battery of validity measures to a group of NIH-funded researchers and research trainees who were diverse in terms of age, years of experience, types of research, and race. The PDR demonstrated adequate reliability (alpha = .84) and parallel form correlation (r = .70). As hypothesized, the PDR was significantly negatively correlated with narcissism, cynicism, moral disengagement, and compliance disengagement; it was not correlated with socially desirable responding. In regression analysis, the strongest predictors of higher PDR scores were low compliance disengagement, speaking English as a native language, conducting clinical research with human subjects, and low levels of narcissism. Given that the PDR was written at an eighth grade reading level to be suitable for use with English as a second language participants and that only one-fourth of items focused on clinical research, further research into the possible roles of culture and research ethics training across specialties is warranted. This initial validity study demonstrates the potential usefulness of the PDR as an educational outcome assessment measure and a research instrument for studies on professionalism and integrity in research.

## Introduction

In this paper, we report on the development and validity of the Professional Decision-Making in Research (PDR) measure, a vignette-based test that examines decision-making strategies used by investigators when confronted with challenging situations in the context of empirical research. This introductory section explains how professionalism in research is related to research ethics, explores the importance of decision-making for professionalism and ethics, identifies impediments to appropriate decision-making, presents compensating strategies for impediments, and explains the need for a new measure of professional decision-making in research.

## Two Core Features of Professionalism

While professionalism has been described in many different ways (van Mook et al. 2009), two key components are commonly mentioned. First, professionalism refers to behaviors that protect the trust that clients and the public place in professionals (ABIM Foundation 2002; Stern and Papadakis 2006; Swick 2000). This is the core idea behind the concept of professional “fiduciary” obligations (Garner 2014). At a minimum, this involves giving priority to professional goals (such as fostering client well-being or discovering new knowledge) over personal interests (such as financial gain or career promotion). Second, professionalism refers to the traits that make individuals “good professionals,” such that they achieve the goals of the profession (Swick 2000; van Mook et al. 2009). In the field of research, the immediate goal is generating new knowledge through systematic activities that allow for replication (Department of Health and Human Services 2005). Thus, professionalism in research requires traits such as being competent, honest, collegial, persistent, and compliant with the rules of funding agencies; such traits are required to generate new knowledge in a systematic manner in modern complex research environments (DuBois 2004; Institute of Medicine 2002).

What is the relationship of professionalism to ethics? We suggest that they are overlapping, but not identical domains. The boundaries between the domains are often circumscribed intuitively and differently depending on who addresses the question. For Aristotelian virtue theorists, the overlap might be nearly complete because the ethical virtues of a researcher are identified by answering the question, “What traits help an individual to achieve the aims (*telos*) of the profession?” (Pellegrino 1995). Thus, consistent with the American Psychological Association’s *Code of Ethics*, which phrases key elements of the code as “Psychologists do *x*,” competence would be a requirement of both professionalism and ethics (Fisher 2003). For Kantian deontologists who focus on universal rights and maxims, the overlap of professionalism and ethics would be relatively incomplete (Sullivan 1994). For example, routinely ignoring the comments of peer reviewers that appear overly harsh might not be unethical, but it could be deemed unprofessional insofar as it is unlikely to lead to funding or the acceptance of publications necessary to thrive as a research professional. In what follows, we assume that professionalism in research encompasses professional ethics as well as any other traits necessary (a) to ensure the trust of research participants and the public or (b) to achieve the aim of producing generalizable knowledge.

## The Importance of Professional Decision-Making

People make thousands of decisions in a day. Although some daily decisions are mundane and relatively inconsequential, many of the decisions made by professionals have significant implications. Researchers’ decisions directly affect the accuracy of data, the protection of research subjects, the quality of collaborations, and the objectivity of peer review, among other things (Shamoo and Resnik 2015; Steneck 2007). When unprofessional decisions are made, suffering can be caused to research subjects, staff, colleagues, institutions, science, and the broader society (De Cremer and van Dijk 2003; Olson 2010). Furthermore, professionals’ decisions, and the thinking patterns underlying them, affect their own work productivity, career success, and overall well-being (Dolbier et al. 2001; Marques-Quinteiro and Curral 2012; Roche et al. 2014; Spreitzer et al. 2005).

At times, decision-making as a professional is straightforward—the “right” or best option is clear. At other times, however, professional decision-making can require navigating complex, dynamic circumstances, considering diverse constituencies, wrestling with conflicting ethical principles, and selecting from multiple options—options that do not present a clear, optimal choice (Mumford et al. 2007; Weick et al. 2005; Thiel et al. 2012).

Given that decision-making is a central hallmark of human life, decision-making processes have been the focus of numerous research studies across many fields (e.g., psychology, organizational behavior, economics, marketing, neuroscience, and medicine) (Breiter et al. 2001; Gino et al. 2013; Greene and Haidt 2002; Hahn et al. 2014; Messick and Bazerman 1996; Milkman et al. 2008; Oppenheimer and Kelso 2015; Stone and Moskowitz 2011; Yang et al. 2013). The findings from this work provide several robust implications for professional decision-making and how professionals might strive to make the best decisions (Bazerman 2001; Bornstein and Emler 2001).

## Impediments to Professional Decision-Making

Several characteristics of situations and problems commonly encountered by professionals threaten the quality of their decision-making. Professionals are particularly at risk for flawed decision-making when they face new, unfamiliar circumstances. In unfamiliar situations, an individual may misinterpret rules, norms, and cues and fail to consider the full range of alternatives and possible outcomes (Palazzo et al. 2012; Thiel et al. 2012).

In addition, professionals routinely encounter complexity in their work. This presents an obstacle because complex situations present ambiguous or conflicting rules, goals, or stakeholder interests and can involve missing, incomplete, and complicated facts. Thus, it is a considerable challenge for professionals to analyze the facts of a situation fully, accurately, and fairly and arrive at a prudent decision (Bazerman and Moore 2013; De Cremer and van Dijk 2003). While professionals expect to encounter new and complex problems in their work, even the most experienced, intelligent, and well-intentioned individuals are subject to the natural limitations of human decision-making that arise from how the brain processes information (Campbell et al. 2009; Hammond et al. 1998; Bazerman and Gino 2012). For example, people tend to focus upon information that conforms to their existing beliefs and preconceived conclusions, ignoring contradictory evidence (Kunda 1990; Nickerson 1998). This confirmation bias manifests in decisions about political candidates, purchases, selecting employees, and new business ventures (Bazerman and Moore 2013). Furthermore, individuals tend to be overconfident in their reasoning ability, exacerbating biases and minimizing the use of strategies that might offset biases (Boiney et al. 1997; Pronin et al. 2004). Kahneman (2003) discusses many other errors in judgment (often called biases or cognitive distortions) that have been identified through empirical studies.

When professional relationships and roles present competing interests, professionals are at risk for self-serving biases that contribute to poor decision-making (Dana and Loewenstein 2003). Many professional organizations and societies apply policies and rules as structural safeguards against the influence of conflicts of interest (AAMC-AAU 2008; DuBois et al. 2013). It is important to note that self-serving and other biases typically operate outside an individual’s conscious awareness (Epley and Caruso 2004; Haidt 2001; Moore and Loewenstein 2004). Wanting to make the “right” or “best” choice does not ensure it will happen; people are not fully rational decision makers (Kahneman et al. 2011; Tenbrunsel et al. 2010).

In addition to environmental factors such as conflicting interests or novel and complex situations, emotions and stress influence reasoning processes and can impinge upon effective decision-making (Angie et al. 2011; Gross 2013). Professionals are at particular risk when negative emotions are heightened and they remain unregulated (Thiel et al. 2011). High stress presents a similar challenge (Selart and Johansen 2010; van Zyl and Lazenby 2002). Notably, even in the absence of a situation-specific stressor, generalized stress and negative emotions can affect decision-making (Andrade and Ariely 2009). Emotions and stress exhaust cognitive and emotional resources, leading to hasty, even unethical decisions (and actions) and to significant reductions in the capacity to filter out the effects of biases (Gino et al. 2011; Mead et al. 2009).

A final risk for professional decision-making exists when an individual falls into patterns of distorted cognitions that go beyond the ordinary human biases described above. Self-serving cognitive distortions include, for instance, blaming others, assuming the worst, euphemistic labeling, or minimizing the consequences of certain decisions (Tenbrunsel and Messick 2004; Barriga and Gibbs 2006). This thinking can lead to defensive, retaliatory, and other undermining behaviors, including decisions and behaviors marked by moral disengagement (Bandura 2002; Moore et al. 2012). Different factors can occasion such patterns, including contextual cues (e.g., competition) and personality characteristics (e.g., cynicism and inflated self-confidence) (Detert et al. 2008). Even though these patterns do not serve an individual well, they are difficult to abandon once they have become habitual (Alexander et al. 2010).

In sum, although professionals’ usual decision-making approaches often serve them well, the challenges and risk factors described above can impact effective decision-making. It is important to note, however, that our points are not meant to imply that emotions and intuition are always problematic. Indeed, they can play an adaptive role in human functioning (Gross 1999). Furthermore, cognitive errors arise from mental short-cuts that help us simplify and understand reality and act accordingly. As Sunstein (2005) suggests, heuristics or short-cuts such as “punish, and do not reward, betrayals of trust” often work fine in navigating moral situations (Sunstein 2005). However, such heuristics can also prove too simple to serve us well in complex situations. Thus, professionals can benefit from a structured approach to navigating professional decision-making.

## Compensating Strategies

A structured approach to professional decision-making can help professionals learn to employ strategies that activate thinking associated with higher quality, more ethical choices that offset biases, harmful emotions, stress, and destructive thought patterns (Neck and Manz 1992, 1996; Thiel et al. 2012; Bazerman and Moore 2013). Awareness of the challenges and risk factors alone may help, but a structured decision making aid offers a proactive, systematic tool (Bornstein and Emler 2001). In addition to minimizing mistakes and potential decisional weaknesses, a structured approach affords professionals a mechanism for self-reflection. This allows professionals to leverage their strengths and grow from their experiences (Ashford and DeRue 2012; Sonenshein et al. 2013).

When confronted with a new, unfamiliar, or complex situation, several strategies support analysis of the situation and evaluation of potential decisions. Seemingly obvious, but neglected strategies include seeking additional information and speaking to others who can challenge dysfunctional assumptions (Sonenshein 2007). Such information search strategies allow overlooked, discounted, or misinterpreted information to be uncovered (Mumford et al. 2007). Other assumption testing strategies, such as reflecting on personal goals, further assist with these aims (Reynolds 2006). Individuals should also consider as many options and potential outcomes as possible, especially focusing on likely consequences of a decision (Hsee et al. 1999; Stenmark et al. 2011; Watley and May 2004). Ultimately, these compensatory strategies increase the likelihood of making appropriate connections between facts, contextual cues, alternatives, and likely outcomes (Thiel et al. 2012).

A key strategy when faced with competing interests that might engender self-serving biases is shifting from fast, reactive processing to slower, deliberative processes (Sonenshein 2007). Strategies aimed at examining one’s motivations and requesting feedback from others induce such a shift (DuBois et al. 2013). In the case of heightened negative emotions and stress, primary compensatory strategies include emotion and stress management. For instance, emotion management involves identifying emotions and then responding to them with techniques such as reappraisal or relaxation or combinations of such strategies (Barrett et al. 2001; Searle 2008; Sidle 2008; Unsworth and Mason 2012). When stress is high or emotions are charged, important secondary strategies may include realistically assessing the situation (e.g., causes, alternatives, potential consequences, roles, and responsibilities) and seeking help (Bazerman et al. 2011; Hülsheger et al. 2013; Searle et al. 2001; Thiel et al. 2011).

Compensatory strategies can also aid in diminishing and overcoming cognitive distortions. Here, it is essential to pursue greater awareness and understanding through testing assumptions and motives, especially comparing one’s viewpoint to the perspectives of others (Taylor et al. 2011). Additionally, it is important to seek input from others, in particular inviting corrective feedback that might challenge one’s mindset (Hauer and Kogan 2012; Sommer and Kulkarni 2012; van der Rijt et al. 2012). Emotion and stress management strategies may be critically important secondary strategies when stress or emotions serve as triggers for cognition distortions. Growing evidence for mindfulness and meditative training programs suggests these, too, are promising techniques for shifting perceptions, improving work-related stress, and improving overall functioning (Hawkins 2003; Shonin et al. 2014; Van Gordon et al. 2014).

Of course, a decision aid is only useful if it is recalled and applied when necessary. Thus, it is important for decision strategies to be structured in a practically useful, memorable fashion. The SMART Strategies™ developed by the Professionalism and Integrity in Research Program (PI Program) illustrates such a tool (DuBois 2014). The PI Program was developed with funding from the National Institutes of Health (NIH) to provide education for researchers who have had compliance or research integrity challenges within their labs (http://integrityprogram.org). Drawing from the literature referenced above, the PI Program developers packaged compensatory strategies for professional decision-making according to five domains: Seek help; Manage your emotions; Anticipate consequences; Recognize rules and context; and Test your assumptions and motives. Table 1 summarizes the link between professional challenges, compensatory strategies, and the discrete facets of the SMART Strategies™. This approach has been used successfully with participants in the PI Program. (We expect to publish outcome data in 2016.)

**Table 1:** Linking challenges, strategies, and the SMART™ tool

Professional challenges and risk factors	Compensatory strategies	S.M.A.R.T.™ tool				
		Seek help	Manage emotions	Anticipate consequences	Recognize rules and context	Test assumptions and motives
New, unfamiliar situation or problem	Search for information Analyze causes, norms, and rules Test assumptions Integrate facts, cues, options, and outcomes	✔*		✔	✔*	✔*
Complex situation or problem	Search for information Analyze causes, norms, and rules Test assumptions Integrate facts, cues, options, and outcomes	✔*		✔	✔*	✔*
Relationships or roles with competing interests	Shift from automatic to deliberative processing Examine personal motivations Seeking objective input	✔*	✔	✔	✔	✔*
Heightened negative emotions or stress	Recognize and downgrade emotions Employ stress management techniques Assess the situation or problem realistically Seek outside perspectives and help	✔*	✔*	✔	✔	✔
Cognitive distortions or biased thinking patterns	Reflect on the actual problem or issue at hand Assess the perspective of others Examine personal motivations Seek external guidance, support, and input Employ stress and emotion management techniques	✔*	✔*	✔		✔*

✔*  primary strategy; ✔ secondary strategy

## Rationale for a New Measure

The ethical decision-making measure (EDM) developed by Mumford and colleagues is a well-validated test that focuses on the use of decision-making strategies. It presents realistic professional and ethical problems that include factors such as incomplete knowledge, power discrepancies, and urgency—factors that may interfere with ethical decision-making (Mumford et al. 2006). Thus, the EDM can be considered a measure of professional decision-making in research. Its validity has been supported by studies demonstrating negative correlations with personality traits known to compromise ethical decision-making (such as cynicism and narcissism) and positive correlations with research integrity training that focuses on the use of sense-making strategies (Antes et al. 2007; Kligyte et al. 2008; Mumford et al. 2006). It exists in versions tailored to different scientific specialties, which can increase the accuracy of professional assessments.

Nevertheless, the EDM has several limitations. First, its scenarios are written at a very high reading level (some scenarios exceed a Lexile score of 1400 or a FLESCH grade 13.8). While researchers have a high level of education, high Lexile scores can present difficulties for those with English as a second language (ESL). This poses a problem in the field of research because according to both the National Science Foundation (NSF) and the National Institutes of Health (NIH), approximately half of all post-doctoral fellows working in the US were born outside the US, primarily in non-native English speaking nations (National Science Foundation 2011).

Second, the EDM is time-consuming to complete, making it challenging to use as a pre- and post-test in educational settings. Each version (pre and post) consists of 25 vignette items built around 5 core scenarios and requires test-takers to make 50 choices after reading 200 options (totaling more than 8000 words per form). This makes the EDM particularly problematic when used in educational settings that involve non-native English speakers.

Third, the EDM scoring matrix is based on minimizing harm to self and others, and ratings of the degree to which each of seven decision-making strategies is embodied in each option. While this approach has obvious advantages insofar as one behavior may embody multiple strategies to differing degrees, it makes it difficult to rate options that illustrate the use of one strategy but violate the use of another (e.g., an option that considers consequences and minimizes harms while violating a rule—or vice versa). Thus, even if the EDM has adequate inter-rater reliability on its overarching ethicality score (which classifies responses as high, medium, or low in ethicality), it is not ideally suited to presenting scores focused on the use of specific strategies. We wanted a measure focused on assessing the tacit use of professional problem-solving strategies in research for several reasons:

1. They can be taught in a straightforward manner. It is questionable whether one can teach or inculcate values in a short course on research ethics, but the use of strategies has been taught successfully (Kligyte et al. 2008).
2. The use of problem-solving strategies is a skill that can be applied to many diverse contexts and challenges. They are equally relevant to researchers who work with databases, animals, or humans, or who struggle with privacy rules, animal care protocols, or informed consent procedures.
3. As noted above, the specific problem-solving strategies that we assess have a strong foundation in the empirical literature for enhancing the quality of decision-making.

Finally, the EDM asks investigators to identify “the best” responses. It is meant to assess ethical decision-making as a cognitive task. In contrast, we wanted to develop a measure that explores behavioral intentions by asking what options participants would be “most likely to choose” if they were really in the challenging situation described.

Accordingly, we identified the need for a professional decision-making in research (PDR) measure that (a) is written at a moderate reading difficulty level, (b) requires less time to complete, (c) has items that clearly illustrate a primary decision-making strategy, and (d) focuses on behavioral intentions.

## Methods

### Sampling and Recruitment

For our initial validity study, we recruited a convenience sample of 300 researchers funded by the National Institutes of Health (NIH), working in the United States (US), who were diverse in terms of career stage and reflective of the overall NIH-funded population in terms of gender, age, native language, and field of study. Recruitment was guided by the NIH RePORTER database, which can be sorted by funding mechanisms and identifies the principal investigators of all grants awarded. In order to represent diverse career stages, we targeted individuals who had received at least one of two types of funding: Training grants (T, K) or independent investigator grants (R01 s). In order to increase the number of eligible trainees, we also contacted the principal investigators of institutional research training programs funded through the Clinical and Translational Science Award (CTSA) program with the request that they share our recruitment email with their NIH-funded trainees.

From February through May 2014, potential participants were contacted by email with an invitation to participate in a study that aimed to evaluate a measure of how researchers make professional decisions. We estimated that participation would require 75–120 min and offered $100 in payment. Potential participants received two email reminders at approximately 1 and 3 weeks following initial contact.

### Instrument Development

Instrument development involved writing items to represent the use of SMART Strategies™ (Table 1), testing their Lexile scores to ensure basic readability, and subsequent review aimed at establishing content validity by team members, EDM authors, and cognitive interviewing.

#### Writing Items

In an effort to examine the full range of professional issues in research, we wrote items to represent matters of concern to four oversight offices found in all major research universities: Institutional review boards (IRBs), which oversee human subjects protections; institutional animal care and use committees (IACUCs); research integrity offices (RIOs), which pursue allegations of plagiarism and data fabrication and falsification; and conflict of interest committees (COICs). In addition, we wrote items that pertained to general research practices, such as peer review and interpersonal relationships within research teams. We further developed storylines representing diverse kinds of research: human subjects, animals, biological field research, wet lab science, and data analysis (dry lab).

We produced parallel forms of the PDR (a pre- and a post-test version). Each form consists of 4 scenarios that describe a particular researcher and research project. Each scenario is followed by four vignette-based items that describe a specific challenge faced by the researcher. Each item presents six options and asks participants to provide two different responses, which are described in Table 2, “Sample PDR Item.”

**Table 2:** Sample PDR item

(Scenario) You are a developmental psychologist who studies violence in elementary school children. Your lab includes several PhD students who work as research assistants. Your current project examines whether children with a certain genetic makeup are especially susceptible to the effects of television violence. Part of the project requires obtaining a cheek swab for DNA analysis. After the swab, you interview the children and their parents to determine their television viewing habits. You also observe them in the classroom and interview teachers and principals about the students’ history of violent behavior. Your work depends heavily upon having good relationships with the local schools
(Item) You consider your project’s sensitive nature. You are concerned that parents will be reluctant to allow their children to participate. You are writing the informed consent form and fear that too much detail might discourage participation. Consider the following options.
(Instructions) Predict your behavior by identifying the two options you would be most likely to choose if you were really in the challenging situation
(Response options) 1. Tell the institutional review board (IRB) and parents that you are studying the influence of television; downplay the role of violence. (Less professional)
2. Along with the consent form, send a handout approved by your IRB that addresses parents’ common questions and concerns. (More professional, Anticipating Consequences/Recognizing rules)
3. Ask colleagues who have successfully recruited students to similar research how they deal with this challenge. (More professional, Seeking help)
4. Ask yourself what is the worst thing that could happen in this study to explore whether the risks are really as trivial as you think. (More professional, Testing assumptions)
5. Mention only those risks that are likely to occur. (Less professional)
6. Disclose every risk in the consent form, but send a follow up letter to parents who do not give permission explaining that the risks are small. (Less professional)

Of the six options that follow each item, three were written to represent “less” professionally effective choices, and three were written to represent “more” professionally effective choices. Less effective choices violate at least one of the five professional decision-making strategies presented in Table 1; more effective choices illustrate use of one of the strategies. The number of options illustrating each strategy varied according to the strategy illustrated. For example, we had fewer managing emotion options than seeking help options, because we wanted options to fit naturally with the vignettes. A managing emotions option was presented only if the vignette mentioned an emotional dimension such as “You feel upset and worried that this accusation will blemish your reputation.” Nevertheless, the parallel forms contain identical numbers of options representing each of the strategies (13 seeking help, 6 managing emotions, 17 anticipating consequences/recognizing rules, and 12 testing assumptions).

#### Lexile Analysis

All scenarios and item stems were submitted to Lexile analysis following the NIH PROMIS guidelines (National Institutes of Health 2012). The overall PDR (combining both forms) had a mean Lexile score of 930 with a range from 720 to 1100 for individual items. To provide a frame of reference, eighth graders in the US demonstrate an interquartile range from 805 to 1100 (https://www.lexile.com/about-lexile/grade-equivalent/grade-equivalent-chart/).

#### Establishing Content Validity of Items and Codes

The first author drafted all items. Approximately half of the PDR scenarios are based on EDM storylines; the other scenarios are novel. Content validity was established through three methods that are described more fully below: (1) meetings of the authorship team, which was comprised of four individuals with significant expertise in research and research ethics; (2) review by the EDM authors’ research team; and (3) cognitive interviews conducted by the project coordinator with a cohort of 6 experts in research, research ethics, and compliance.

#### Authorship Team Meetings

The first four authors hold doctoral degrees in psychology; all have served on IRBs and as principal investigators of federally-funded grants; one directs a research ethics center, one serves as a chief research officer, and two have chaired an IRB. This team of four met face-to-face to review draft items (vignettes and options) on three occasions to refine items for clarity and content validity. Review of the response options focused on revising responses until consensus was achieved that each represented its intended strategy (e.g., seeking help or managing emotions), and each “less” effective choice in fact violated a strategy while remaining plausible as a choice.

#### EDM Team Review

Next, the members of the research team that developed the EDM reviewed all items and the coding system. They were asked to code items as “more” or “less” professional, and to consider whether items were plausible. Items were revised again based on a report from the EDM team that suggested the need for a greater number of plausible distractors to increase variance; this led the authors to re-write some of the “less” professional options to make them less obviously unprofessional.

#### Cognitive Interviewing

Finally, using a guide developed by the first four authors, the research coordinator conducted cognitive interviews with six individuals in a variety of professional backgrounds and levels of experience, including compliance and IRB professionals (Willis 2006). Two of the individuals were born abroad and spoke English as a second language. These interviews led to some changes in wording for selected items.

Finally, prior to distributing the PDR to the target sample, it was sent to a small pilot sample of 10 participants to ensure that the survey system was working properly and the survey contained no critical errors. The many layers of feedback and revision resulted in a tool that was deemed clear and understandable with items that are relevant and realistic.

### Construct Validation Measures

Construct validation focused on identifying constructs that prior research suggests should correlate negatively with professional decision-making, identifying appropriate tests of these constructs, and testing the correlations of PDM scores with these test scores.

#### Narcissism

Past research found that narcissism is negatively correlated with professional practices (Antes et al. 2007). We assessed narcissism using the NPI-16, a 16-item test with strong reliability and validity (Ames et al. 2006; Raskin and Terry 1988).

#### Cynicism

Based on prior data indicating that higher levels of cynicism are correlated with lower levels of ethical decision-making (Mumford et al. 2006), we expected a valid measure of PDR to correlate negatively with cynicism. We assessed levels of cynicism using the 11-item Global Cynicism Scale (GCS) which has demonstrated validity and reliability (Turner and Valentine 2001).

#### Moral Disengagement

Bandura and colleagues have described moral disengagement as “psychological maneuvers by which moral self-sanctions can be disengaged” from unethical conduct (Bandura 1999). Such maneuvers include the use of cognitive distortions such as euphemistic labeling, victim blaming, and minimizing harms. We hypothesized that individuals who were high in moral disengagement would score lower on the PDR because they are at risk of distorted perceptions of consequences, others, and rules. We assessed levels of moral disengagement using the Propensity for Moral Disengagement Scale (MDS), an 8-item test that has demonstrated validity and reliability (Moore et al. 2012).

#### Compliance Disengagement

Because the MDS focuses on disengagement from general moral sanctions, we investigated the relationship of PDR scores to disengagement from research compliance using a new measure initially validated with the same group of participants. The How I Think about Research (HIT-Res) scale is a 45-item test that is modeled on the How I Think (HIT) test. The HIT has demonstrated excellent validity and reliability in multiple studies assessing levels of self-serving cognitive distortions (Stams et al. 2006). The HIT-Res changed the behavioral referents of items from antisocial behaviors (such as lying and stealing) to behaviors that deviate from research integrity or compliance. The HIT-Res demonstrated excellent construct validity and reliability in this study (DuBois et al. 2015).

#### Social Desirability

We included the 13-item Marlowe-Crowne Social Desirability Scale (SDS) (Reynolds 1982) to determine and control for the degree to which PDR scores might be associated with socially desirable response sets.

### Procedures

All tests were uploaded into Qualtrics survey software. A link to the online survey (test battery) was sent to potential participants by email with an invitation to participate. The informed consent form comprised the first four pages of the survey. We used the forced-choice option to ensure complete data. Data from a participant were used only when the entire survey was completed. All participants completed both forms of the PDR as well as all validation measures and the demographic survey.

### Analytical Approach

We examined the reliability of the PDR in this study by generating overall alpha values and by examining the correlation of the two parallel forms (split half reliability). We then generated descriptive data for the PDR (mean, standard deviation, range). We awarded one point for each item on which the participant’s two choices *both* illustrated use of a SMART strategy, yielding a total score range 0-16 for each parallel form. This scoring approach was theoretically most consistent with our goals, because selecting even one “less professional” option can lead to harm. Additionally, when given two choices for each item, picking just one option that illustrates the use of SMART Strategies™ can be accomplished through random guessing, which we did not want to reward. We additionally generated four subscores to reflect how frequently a participant selected a specific SMART Strategy™ when presented with it and called these “strategy preference profile” scores. Because the PDR cannot be factor analyzed (given its “pick 2” format), we did not test the extent to which the subscales functioned as anything other than a priori constructs (validated through expert ratings).

We examined the correlation of social desirability with the PDR to determine if it should be used as a control variable in the analyses. Correlations between the PDR and cynicism, narcissism, compliance disengagement, and moral disengagement were then examined. Next, we examined the association of demographic variables such as years of experience conducting research and English as a second language (ESL) with PDR scores. Variables with statistically significant effects were considered for inclusion in a forward selection regression analysis to identify which variables independently predicted PDR scores. Finally, we conducted a cluster analysis to examine whether test-takers clustered into distinct groups. Specifically, we wanted to determine whether the PDR would identify outlier groups, as this would increase its value as an outcome measure for training programs.

### Research Ethics

The Institutional Review Board at Washington University in St. Louis approved the study. The survey included a 4-page consent form. Participants indicated consent by clicking a button to proceed to the test items.

## Results

We received 300 completed test batteries from NIH-funded researchers. Because we used a force-choice approach, we had no missing data on any of the test instruments.

### Basic Descriptive Statistics and Reliability

The PDR demonstrated adequate reliability with a Cronbach’s alpha reliability coefficient of .84 and a parallel forms correlation of .70. We observed a range of 4–32 “items correct” out of a possible total of 32 with a mean score of 26.37 (SD = 4.57). The mean score indicates that participants’ two choices on an item *both* illustrated the use of SMART Strategies™ approximately 81 % of the time. Mean scores on the parallel forms were nearly identical (*M* = 13.04 and 13.33 respectively, SD = 2.31 and 2.65 respectively).

### Demographics

Table 3 presents the demographics of the sample and the results of ANOVAs and t-tests, which established whether PDR scores differed significantly between demographic groups. The sample included a wide distribution of ages, years of experience doing research, and career stage (with 51 % designating themselves as trainees, which included pre-doctoral and post-doctoral fellows, and early career development scholars). The sample also included diverse kinds of researchers. Researchers could designate their activities using more than one category. The largest groups were clinical (46 %), wet lab (44 %), animal (37 %), and social-behavioral (32 %) investigators. Seventy-eight percent of participants designated themselves as White, 16 % as Asian, and 6 % as African American; 7 % identified their ethnicity as Hispanic, the remainder (per NIH reporting policy) as non-Hispanic. Sixteen percent spoke English as a second language; of these, 48 % identified as White and 48 % identified as Asian. The sample was 57 % female and 43 % male.

**Table 3:** Demographics and differences among subgroups

Variable	N	%	PDR mean	SD	F/t value	*p* value
*Age*						
20–29	93	31	26.32	4.97	F = .31	.86
30–39	134	45	26.25	4.33		
40–49	52	17	26.40	4.73		
>50	21	7	27.29	3.90		
*Gender*						
Male	128	43	25.59	5.31	t = −2.57	.01
Female	172	57	26.95	3.84		
*Years doing research*						
0–5	104	35	26.00	5.48	F = .57	.64
6–10	119	40	26.78	3.87		
11–20	57	19	26.25	4.18		
20+	20	7	26.25	4.38		
*Funding status: trainee*						
Yes	152	51	26.55	4.75	t = −.69	.49
No	148	49	26.19	4.38		
*Current research funded by pharmaceutical, medical device or other health care industry*						
Yes	40	13	26.78	4.76	t = .58	.57
No	260	27	26.31	4.54		
*Human subjects research: social or behavioral*						
Yes	96	32	27.50	3.89	t = −2.97	.003
No	204	68	25.84	4.77		
*Human subjects research: clinical*						
Yes	138	46	27.29	3.64	t = −3.26	.001
No	162	54	25.59	5.11		
*Animal research*						
Yes	111	37	25.39	4.83	t = 2.81	.005
No	189	63	26.95	4.31		
*Dry lab*						
Yes	54	18	26.33	4.60	t = .71	.94
No	246	82	26.38	4.57		
*Wet lab*						
Yes	131	44	25.45	4.46	t = 3.13	.002
No	169	56	27.09	4.53		
*Racial categories*						
White	235	78	26.73	4.30	t = .−2.58	.01
Other	65	22	25.09	5.26		
*Native language*						
Native English speaker	252	84	26.97	4.14	t = 4.56	.000
English as a second language	48	16	23.23	5.39		

### Analysis of Predictor Variables

Table 4 presents mean scores, standard deviations, correlations with the PDR, and multiple regression weights for the construct validation and control variables. The PDR was significantly negatively correlated (*p* < .001) with all of the construct validation measures: narcissism (*r* = −.15), cynicism (*r* = −.26), moral disengagement (*r* = −.32), and compliance disengagement (*r* = −.38). It was not correlated with social desirability (*r* = −.02, *p* = .68); thus, the subsequent regression analysis did not control for social desirability.

**Table 4:** PDR predictor variables: initial effect sizes and multiple regression weights

Scaled variables					
Measure	Mean (scale range)	SD	Pearson’s r	b	β
Moral disengagement (PMD)	1.91 (0–7)	.73	−.32***	–	–
Cynicism (GCS)	3.30 (0–11)	.90	−.26***	–	–
Narcissism (NPI-16)	.26 (0–1)	.18	−.15***	−2.54*	−.10*
Compliance disengagement (HIT-Res)	2.48 (0–6)	.63	−.38***	−2.35***	−.32***
Social desirability (MCSDS)*	6.49 (0–13)	3.04	−.02	n/a	n/a

Dichotomous demographic variables					
	% Yes	% No	*t* test	b	β
White	78	22	−2.58**	–	–
Native English speaker	84	16	4.56***	2.78***	.22***
Clinical research	46	54	−3.26***	1.35**	.15**
Social-behavioral research	32	68	−2.97**	–	–
Animal research	47	63	2.81**	–	–
Wet lab	44	56	−3.13**	–	–
Female	57	43	−2.57**	–	–

* *p* < .05, ** *p* < .01, *** *p*<.001

Interpretation: Pearson’s r and *t* test values indicate the relationship of the variable to PDR scores without controlling for the influence of other variables. The b and beta (β) values indicate whether the variable has independent predictive value after controlling for the influence of other values. SPSS does not report b or beta values for variables that are not statistically significant in a forward entry regression model

Several demographic variables corresponded with statistically higher scores on the PDR: Being female, conducting human subjects research, being White, and speaking English as a native language (which produced the largest difference: *M* = 27.0 vs 23.2, *t* = 4.56, *p* < .001).

Statistically significant correlates (compliance disengagement, moral disengagement, cynicism, narcissism, and demographic variables) were considered for inclusion in a forward entry multiple regression model to predict PDR scores. Table 4 presents the regression results: *R*^2^ = .24, *F*(4, 295) = 22.6, *p* = .001. Four variables were included as predictors of PDR scores: compliance disengagement (β = −.32, *p* < .001), ESL (β = −.22, *p* < .001), conducting clinical research with human subjects (β = .15, *p* < .01), and narcissism (β = −.10, *p* < .05).

### Preference Profiles

Given the PDR’s high overall alpha (.84) and the high correlations of all strategy subscores both within and across parallel forms (.32–.54, *p* < .001), it is reasonable to interpret the test as having one underlying construct: The use of SMART Strategies.™ Nevertheless, the mean scores for items reflecting any one strategy (e.g., the mean “seeking help” score) are more strongly correlated with their own parallel form (.55–.67, *p* < .001) than with any other mean strategy score, and each strategy received strong content validation through expert review of items. The subscores are calculated as a percentage (number of selections/number of appearances of the strategy). The mean percentage scores from our validation sample on the four subscores are seeking help (*M* = .77, SD = .13); managing emotions (*M* = .57, SD = .22); anticipating consequences/recognizing rules (*M* = .79, SD = .13); and testing assumptions (*M* = .67, SD = .16). Thus, we consider subscores to provide meaningful information that reflects the use of each strategy in a manner likely to yield strategy profiles that differ across test-takers.

### Cluster Analysis

A two-step cluster analysis generated 2 clusters. Our sample was split into 99 (33 %) and 201 (67 %) participants, with the former group performing significantly worse on the PDR and all construct validation measures except for narcissism and social desirability. Cluster 1 had a mean PDR of 21.21 (SD 4.23) and cluster 2 had a mean PDR of 28.91 (SD 1.65), t = −22.59, *p* < .001. Similarly, cluster 1’s HIT-Res compliance disengagement scores were significantly higher (worse) than those of cluster 2 (2.76 vs 2.16, t = 5.49, *p* < .001). Thus, the PDR effectively produced two groups in this sample: those who select a less professional option on only 10 % of items and have relatively low levels of compliance disengagement; and those who select a less professional option on 29 % of items with relatively high levels of compliance disengagement.

## Discussion

Study results indicate that we met our initial goals of producing a shorter test of professional decision-making in research written at an 8th grade reading level that demonstrates strong construct validity and reliability (alpha and parallel forms). We expect that the PDR will be useful as an educational assessment measure and as a research instrument.

### Using the PDR in Educational Contexts

The findings in this study indicate that the PDR is likely to be an effective assessment measure for outcomes of research ethics and professionalism training. First, it is sufficiently brief (approximately 30 min) to administer alongside other measures. Second, the PDR has parallel forms with adequate reliability, thus making the PDR suitable for pre- and post-test designs without memory bias for previously seen items. Third, the PDR is written at approximately the eighth-grade reading level, making it suitable for use with native English speakers as well as graduate students and researchers who speak English as a second language. Fourth, the PDR assesses a skill—the use of good professional decision-making strategies (which includes recognizing rules frequently taught in research ethics courses)—that can be taught and learned within the confines of a brief course (Kligyte et al. 2008), unlike such developmental traits as moral reasoning. Finally, the PDR is not correlated with socially desirable responding, making it difficult for participants to “fake high” or respond in ways that they expect their instructors would desire.

The PDR also opens up the possibility of examining the effects of research ethics instruction on those individuals in most need of development. The PDR clearly separated participants into two groups that differed in their professional decision-making. This division of participants into two groups was confirmed by performance on the construct validation measures; for example, the bottom third demonstrated greater disengagement from compliance and higher levels of cynicism. Those in the bottom one-third of test-takers are arguably the individuals of greatest concern for the field of research; the choices that illustrate violations of SMART Strategies™ also typically illustrate breaking rules in science, causing unnecessary harm, or acting impulsively or with incomplete knowledge. Thus, instructors may want to calculate separately the effects of training, such that those scoring in the bottom one-third are considered separately from the whole roster of course participants. We believe that demonstrating improvements with the former group is more important than with the latter.

In Table 5, we contrast the PDR to the EDM, the original test upon which the PDR was largely modeled. As indicated in the table and in our introduction, despite their superficial similarity, the PDR and EDM are quite different in substance. We consider the PDR to be a useful alternative to the EDM, but not a replacement as each provides different information suitable for different contexts.

**Table 5:** Comparison of the ethical decision making (EDM) and professional decision-making (PDM) measures

Readability^a^	Items and choices/book	Time^b^	Primary scores	Secondary scores	Fields	Assesses	Construct validation^d^	Reliability
*EDM*								
Ave: 1260 Upper range: 1400 Grade 15	25 items 50 choices 200 options 8128 words	40–70 min	Ethicality score Range: 0–3	0–3 point sub-scores for: Business practices Data management Professional practices Study conduct	Separate versions for: Health Biological Social Engineering Humanities	Cognitive ability to identify “best” options with a focus on harm reduction to self and others and use of sensemaking strategies	Significant correlation (*p* < .05) with: Narcissism: not significant overall^c^ Cynicism: −.25 Exposure to unethical events: −.32	Alpha: Not reported Split half: .74
*PDM*								
Ave: 930 Upper range: 1100 Grade 8	16 items 32 choices 96 options 3898 words	25–35 min	Professional decision-making Range: 0–16	A preference profile for: Seeking help Managing emotions Anticipating consequences/Recognizing rules Testing assumptions	One version: General empirical research illustrated by diverse fields	Behavioral intentions vis-à-vis options that illustrate either use of or violation of SMART strategies	Significant correlation (*p* < .01) with: Narcissism: −.15 Cynicism: −.26 Compliance disengagement: −.38	Alpha: .84 Split half: .70

^a^Readability calculated using Lexile analysis of item stems only following NIH PROMIS guidelines (National Institutes of Health 2012)

^b^Estimated average time requirements for diverse group of participants

^c^Only the Professional Practices subscore of the EDM was negatively correlated with narcissism (*r* = −.22, *p* < .05)

^d^This reporting of construct validation of the EDM is partial and emphasizes areas of overlap with the PDR. The EDM has been validated in further studies using multiple outcomes

### The Value of the PDR as a Research Instrument

Apart from its use in assessing outcomes of research training programs, the PDR demonstrated its potential usefulness as a tool for conducting research on professional decision-making. While several trait and demographic variables were significantly correlated with higher PDR scores, four variables demonstrated independent predictive value in a regression analysis: engagement with compliance-related issues, speaking English as a native language, conducting clinical research, and exhibiting lower levels of narcissism.

In this study of NIH-funded researchers, ESL participants were evenly divided by race with 48 % identifying as White and 48 % as Asian. This raises the possibility that cultural variation in what is considered to be professionally adaptive may explain differences in PDR scores more than English fluency per se, especially considering that the PDR was intentionally written at the 8^th^ grade reading level and nearly all of our test-takers held PhD or MD degrees and all worked in the US. We believe this finding merits further investigation into the possible effects of language, culture, and acculturation on professional decision-making in research. Related to this line of investigation is exploration of the role of research experience. It is notable that experience—measured both as a dichotomous variable (trainee vs. non-trainee) and as a continuous variable (years of experience conducting research)—had no association with PDR scores. This could be due to the nature of the trainees in this study—they were all in NIH-funded training programs, which require training in the responsible conduct of research and close work with mentors, typically in research-intensive environments. But it also indicates that other factors are stronger determinants of PDR than experience alone.

Conducting clinical research with human subjects rather than other forms of research was associated with higher PDR scores. We do not think this is due to the nature of the items, as vignettes were written to represent research across various disciplines and only 1 in 4 vignettes in each of the parallel forms illustrates clinical research with humans. Rather, it is possible (a) that human subjects researchers receive more frequent and longer training than other researchers, or (b) that clinical research effectively puts a human face on matters of research professionalism and compliance, increasing the importance attached to such matters. We believe this finding merits further investigation into the role of research ethics education and values in research on professional decision-making in research.

## References

[CR1] AAMC-AAU (2008). Protecting patients, preserving integrity, advancing health: Accelerating the implementation of COI policies in human subjects research. *AAMC* (pp. 1–87). Washington, DC: AAMC-AAU.

[CR2] Alexander GC, Guerrero C, Park H, Humensky J, Loewenstein G (2010). Brief report: Physician narcissism, ego threats, and confidence in the face of uncertainty. Journal of Applied Social Psychology.

[CR3] Ames DR, Rose P, Anderson CP (2006). The NPI-16 as a short measure of narcissism. Journal of Research in Personality.

[CR4] Andrade EB, Ariely D (2009). The enduring impact of transient emotions on decision making. Organizational Behavior and Human Decision Processes.

[CR5] Angie AD, Connelly S, Waples EP, Kligyte V (2011). The influence of discrete emotions on judgement and decision-making: A meta-analytic review. Cognition and Emotion.

[CR6] Antes AL, Brown RP, Murphy ST, Waples EP, Mumford MD, Connelly S (2007). Personality and ethical decision-making in research: The role of perceptions of self and others. Journal of Empirical Research on Human Research Ethics.

[CR7] Ashford SJ, DeRue DS (2012). Developing as a leader. Organizational Dynamics.

[CR8] Bandura A (1999). Moral disengagement in the perpetration of inhumanities. Personality and Social Psychology Review.

[CR9] Bandura A (2002). Selective moral disengagement in the exercise of moral agency. Journal of Moral Education.

[CR10] Barrett LF, Gross J, Christensen TC, Benvenuto M (2001). Knowing what you’re feeling and knowing what to do about it: Mapping the relation between emotion differentiation and emotion regulation. Cognition and Emotion.

[CR11] Barriga AQ, Gibbs JC (2006). Measuring cognitive distortion in antisocial youth: Development and preliminary validation of the ‘How I Think’ Questionnnaire. Aggressive Behavior.

[CR12] Bazerman MH (2001). The study of ‘real’ decision making. Journal of Behavioral Decision Making.

[CR13] Bazerman MH, Gino F (2012). Behavioral ethics: Toward a deeper understanding of moral judgment and dishonesty. Annual Review of Law and Social Science.

[CR14] Bazerman MH, Gino F, Shu LSL, Tsay CJ (2011). Joint evaluation as a real-world tool for managing emotional assessments of morality. Emotion Review.

[CR15] Bazerman MH, Moore DA (2013). Judgment in managerial decision making.

[CR16] Boiney LG, Kennedy J, Nye P (1997). Instrumental bias in motivated reasoning: More when more is needed. Organizational Behavior and Human Decision Processes.

[CR17] Bornstein BH, Emler AC (2001). Rationality in medical decision making: A review of the literature on doctors’ decision-making biases. Journal of Evaluation in Clinical Practice.

[CR18] Breiter HC, Aharon I, Kahneman D, Dale A, Shizgal P (2001). Functional imaging of neural responses to expectancy and experience of monetary gains and losses. Neuron.

[CR19] Campbell A, Whitehead J, Finkelstein S (2009). Why good leaders make bad decisions. Harvard Business Review.

[CR20] Dana J, Loewenstein G (2003). A social science perspective on gifts to physicians from industry. Journal of the American Medical Association.

[CR21] De Cremer D, van Dijk E (2003). Fairness and ethics in social decision making. Social Justice Research.

[CR22] Department of Health and Human Services (2005). Code of federal regulations. Protection of human subjects. Title 45 public welfare, part 46. Washington, DC: Government Printing Office.

[CR23] Detert JR, Trevino LK, Sweitzer VL (2008). Moral disengagement in ethical decision making: A study of antecedents and outcomes. Journal of Applied Psychology.

[CR24] Dolbier CL, Soderstrom M, Steinhardt MA (2001). The relationships between self-leadership and enhanced psychological, health, and work outcomes. The Journal of Psychology: Interdisciplinary and Applied.

[CR25] DuBois JM (2004). Is compliance a professional virtue of researchers? Reflections on promoting the responsible conduct of research. Ethics and Behavior.

[CR26] DuBois JM (2014). Strategies for professional decision making: The SMART approach.

[CR27] DuBois, J. M., Chibnall, J. E., & Gibbs, J. C. (2015). Compliance disengagement in research: Development and validation of a new measure. *Manuscript submitted for publication*. 10.1007/s11948-015-9681-x PMC4996885 26174934

[CR28] DuBois JM, Kraus E, Mikulec A, Cruz S, Bakanas E (2013). A humble task: Restoring virtue in medicine in an age of conflicted interests. Academic Medicine.

[CR29] Epley N, Caruso EM (2004). Egocentric ethics. Social Justice Research.

[CR30] Fisher CB (2003). Decoding the ethics code: A practical guide for psychologists.

[CR31] Foundation ABIM (2002). Medical professionalism in the new millennium: A physician charter. Annals of Internal Medicine.

[CR32] Garner BA (2014). Black’s law dictionary.

[CR33] Gino F, Ayal S, Ariely D (2013). Self-serving altruism? The lure of unethical actions that benefit others. Journal of Economic Behavior & Organization.

[CR34] Gino F, Schweitzer ME, Mead NL, Ariely D (2011). Unable to resist temptation: How self-control depletion promotes unethical behavior. Organizational Behavior and Human Decision Processes.

[CR35] Greene J, Haidt J (2002). How (and where) does moral judgment work?. Trends in Cognitive Sciences.

[CR36] Gross JJ (1999). Emotion regulation: Past, present, future. Cognition and Emotion.

[CR37] Gross JJ (2013). Emotion regulation: Taking stock and moving forward. Emotion.

[CR38] Hahn T, Preuss L, Pinkse J, Figge F (2014). Cognitive frames in corporate sustainability: Managerial sensemaking with paradoxical and business case frames. Academy of Management Review.

[CR39] Haidt J (2001). The emotional dog and its rational tail: A social intuitionist approach to moral judgment. Psychological Review.

[CR40] Hammond JS, Keeney RL, Raiffa H (1998). The hidden traps in decision making. Harvard Business Review.

[CR41] Hauer KE, Kogan JR (2012). Realising the potential value of feedback. [Article]. Medical Education.

[CR42] Hawkins MA (2003). Section I: Theory and review. Effectiveness of the transcendental meditation program in criminal rehabilitation and substance abuse recovery: A review of the research. Journal of Offender Rehabilitation.

[CR43] Hsee CK, Loewenstein GF, Blount S, Bazerman MH (1999). Preference reversals between joint and separate evaluations of options: A review and theoretical analysis. Psychological Bulletin.

[CR44] Hülsheger UR, Alberts HJEM, Feinholdt A, Lang JWB (2013). Benefits of mindfulness at work: The role of mindfulness in emotion regulation, emotional exhaustion, and job satisfaction. Journal of Applied Psychology.

[CR45] Institute of Medicine, N. R. C. (2002). Integrity in scientific research: Creating an environment that promotes responsible conduct.

[CR46] Kahneman D (2003). A perspective on judgment and choice—Mapping bounded rationality. American Psychologist.

[CR47] Kahneman D, Lovallo D, Sibony O (2011). Before you make that big decision. Harvard Business Review.

[CR48] Kligyte V, Marcy RT, Waples EP, Sevier ST, Godfrey ES, Mumford MD (2008). Application of a sensemaking approach to ethics training in the physical sciences and engineering. Science and Engineering Ethics.

[CR49] Kunda Z (1990). The case for motivated reasoning. Psychological Bulletin.

[CR50] Marques-Quinteiro P, Curral LA (2012). Goal orientation and work role performance: Predicting adaptive and proactive work role performance through self-leadership strategies. The Journal of Psychology: Interdisciplinary and Applied.

[CR51] Mead NL, Baumeister RF, Gino F, Schweitzer ME, Ariely D (2009). Too tired to tell the truth: self-control resource depletion and dishonesty. Journal of Experimental Social Psychology.

[CR52] Messick DM, Bazerman MH (1996). Ethical leadership and the psychology of decision making. Sloan Management Review.

[CR53] Milkman KL, Rogers T, Bazerman MH (2008). Harnessing our inner angels and demons what we have learned about want/should conflicts and how that knowledge can help us reduce short-sighted decision making. Perspectives on Psychological Science.

[CR54] Moore C, Detert JR, Trevino LK, Baker VL, Mayer D (2012). Why employees do bad things: Moral disengagement and unethical organizational behavior. Personnel Psychology.

[CR55] Moore DA, Loewenstein G (2004). Self-interest, automaticity, and the psychology of conflict of interest. Social Justice Research.

[CR56] Mumford MD, Devenport LD, Brown RP, Connelly S, Murphy ST, Hill JH (2006). Validation of ethical decision making measures: Evidence for a new set of measures. Ethics and Behavior.

[CR57] Mumford MD, Friedrich TL, Caughron JJ, Byrne CL (2007). Leader cognition in real-world settings: How do leaders think about crises?. Leadership Quarterly.

[CR58] National Institutes of Health (2012). PROMIS—Instrument development and psychometric evaluation scientific standards.

[CR59] National Science Foundation (2011). Graduate students and postdoctorates in science and engineering: Fall 2011. http://www.nsf.gov/statistics/nsf13331/content.cfm?pub_id=4290&id=2. Accessed 2 April 2014.

[CR60] Neck CP, Manz CC (1992). Thought self-leadership: The influence of self-talk and mental imagery on performance. Journal of Organizational Behavior.

[CR61] Neck CP, Manz CC (1996). Thought self-leadership: The impact of mental strategies training on employee cognition, behavior, and affect. Journal of Organizational Behavior.

[CR62] Nickerson RS (1998). Confirmation bias: A ubiquitous phenomenon in many guises. Review of General Psychology.

[CR63] Olson LE (2010). Developing a framework for assessing responsible conduct of research education programs. Science and Engineering Ethics.

[CR64] Oppenheimer DM, Kelso E (2015). Information processing as a paradigm for decision making. Annual Review of Psychology.

[CR65] Palazzo G, Krings F, Hoffrage U (2012). Ethical blindness. Journal of Business Ethics.

[CR66] Pellegrino ED (1995). Toward a virtue-based normative ethics for the health professions. Kennedy Institute of Ethics Journal.

[CR67] Pronin E, Gilovich T, Ross L (2004). Objectivity in the eye of the beholder: Divergent perceptions of bias in self versus others. Psychological Review.

[CR68] Raskin R, Terry H (1988). A principal-components analysis of the narcissistic personality inventory and further evidence of its construct validity. Journal of Personality and Social Psychology.

[CR69] Reynolds WM (1982). Development of reliable and valid short forms of the Marlowe-Crowne Social Desirability Scale. Journal of Clinical Psychology.

[CR70] Reynolds SJ (2006). A neurocognitive model of the ethical decision-making process: implications for study and practice. Journal of Applied Psychology.

[CR71] Roche M, Haar JM, Luthans F (2014). The role of mindfulness and psychological capital on the well-being of leaders. Journal of Occupational Health Psychology.

[CR72] Searle BJ (2008). Does personal initiative training work as a stress management intervention?. Journal of Occupational Health Psychology.

[CR73] Searle BJ, Bright JEH, Bochner S (2001). Helping people to sort it out: The role of social support in the Job strain model. Work & Stress.

[CR74] Selart M, Johansen ST (2010). Ethical decision making in organizations: The role of leadership stress. Journal of Business Ethics.

[CR75] Shamoo AE, Resnik DB (2015). Responsible conduct of research.

[CR76] Shonin E, Van Gordon W, Dunn TJ, Singh NN, Griffiths MD (2014). Meditation awareness training (MAT) for work-related wellbeing and job performance: A randomised controlled trial. International Journal of Mental Health and Addiction.

[CR77] Sidle SD (2008). Workplace stress management interventions: What works best?. The Academy of Management Perspectives.

[CR78] Sommer KL, Kulkarni M (2012). Does constructive performance feedback improve citizenship intentions and job satisfaction? The roles of perceived opportunities for advancement, respect, and mood. Human Resource Development Quarterly.

[CR79] Sonenshein S (2007). The role of construction, intuition, and justification in responding to ethical issues at work: The sensemaking-intuition model. Academy of Management Review.

[CR80] Sonenshein S, Dutton JE, Grant AM, Spreitzer GM, Sutcliffe KM (2013). Growing at work: Employees’ interpretations of progressive self-change in organizations. Organization Science.

[CR81] Spreitzer G, Sutcliffe K, Dutton J, Sonenshein S, Grant AM (2005). A socially embedded model of thriving at work. Organization Science.

[CR82] Stams GJ, Brugman D, Dekovic M, van Rosmalen L, van der Laan P, Gibbs JC (2006). The moral judgment of juvenile delinquents: A meta-analysis. Journal of Abnormal Child Psychology.

[CR83] Steneck, N. H. (2007). ORI Introduction to the responsible conduct of research. (Vol. Revised): US Government Printing Office.

[CR84] Stenmark CK, Antes AL, Thiel CE, Caughron JJ, Wang XQ, Mumford MD (2011). Consequences identification in forecasting and ethical decision-making. Journal of Empirical Research on Human Research Ethics.

[CR85] Stern DT, Papadakis M (2006). The developing physician—Becoming a professional. New England Journal of Medicine.

[CR86] Stone J, Moskowitz GB (2011). Non-conscious bias in medical decision making: What can be done to reduce it?. Medical Education.

[CR87] Sullivan R (1994). An Introduction to Kant’s ethics.

[CR88] Sunstein CR (2005). Moral heuristics. Behavioral and Brain Sciences.

[CR89] Swick HM (2000). Toward a normative definition of medical professionalism. Academic Medicine.

[CR90] Taylor C, Farver C, Stoller JK (2011). Perspective: Can emotional intelligence training serve as an alternative approach to teaching professionalism to residents?. Academic Medicine.

[CR91] Tenbrunsel AE, Diekmann KA, Wade-Benzoni KA, Bazerman MH (2010). The ethical mirage: A temporal explanation as to why we are not as ethical as we think we are. Research in Organizational Behavior: An Annual Series of Analytical Essays and Critical Reviews.

[CR92] Tenbrunsel AE, Messick DM (2004). Ethical fading: The role of self-deception in unethical behavior. Social Justice Research.

[CR93] Thiel CE, Bagdasarov Z, Harkrider L, Johnson JF, Mumford MD (2012). Leader ethical decision-making in organizations: Strategies for sensemaking. Journal of Business Ethics.

[CR94] Thiel CE, Connelly S, Griffith JA (2011). The influence of anger on ethical decision making: Comparison of a primary and secondary appraisal. Ethics and Behavior.

[CR95] Turner JH, Valentine SR (2001). Cynicism as a fundamental dimension of moral decision-making: A scale development. Journal of Business Ethics.

[CR96] Unsworth KL, Mason CM (2012). Help yourself: The mechanisms through which a self-leadership intervention influences strain. Journal of Occupational Health Psychology.

[CR97] van der Rijt J, van de Wiel MWJ, Van den Bossche P, Segers MSR, Gijselaers WH (2012). Contextual antecedents of informal feedback in the workplace. Human Resource Development Quarterly.

[CR98] Van Gordon W, Shonin E, Zangeneh M, Griffiths MD (2014). Work-related mental health and job performance: Can mindfulness help?. International Journal of Mental Health and Addiction.

[CR99] van Mook WN, van Luijk SJ, O’Sullivan H, Wass V, Harm Zwaveling J, Schuwirth LW (2009). The concepts of professionalism and professional behaviour: Conflicts in both definition and learning outcomes. European Journal of Internal Medicine.

[CR100] van Zyl E, Lazenby K (2002). The relation between ethical behaviour and workstress amongst a group of managers working in affirmative action positions. [Article]. Journal of Business Ethics.

[CR101] Watley LD, May DR (2004). Enhancing moral intensity: The roles of personal and consequential information in ethical decision-making. Journal of Business Ethics.

[CR102] Weick KE, Sutcliffe KM, Obstfeld D (2005). Organizing and the process of sensemaking. Organization Science.

[CR103] Yang Y, Vosgerau J, Loewenstein G (2013). Framing influences willingness to pay but not willingness to accept. Journal of Marketing Research.

